# Fragrant rice performances in response to continuous zero-tillage in machine-transplanted double-cropped rice system

**DOI:** 10.1038/s41598-020-65388-0

**Published:** 2020-05-20

**Authors:** Bin Du, Longxin He, Rifang Lai, Haowen Luo, Tantan Zhang, Xiangru Tang

**Affiliations:** 1 Department of Crop Science and Technology, College of Agriculture, South China Agricultural University, Guangzhou, 510642 P.R. China; 2 Scientific Observing and Experimental Station of Crop Cultivation in South China, Ministry of Agriculture, Guangzhou, 510642 P.R. China

**Keywords:** Biochemistry, Plant sciences, Ecology, Environmental sciences

## Abstract

Zero-tillage is one of conservation tillage techniques. In order to investigate the effects of continuous zero-tillage on yield formation and grain 2-acetyl-1-pyrroline (2-AP, key component of fragrant rice aroma) content of fragrant rice, present study was conducted with a six-season field experiment from 2017 to 2019. The conventional tillage (twice puddling with rotary cultivator before transplanting) was set as control (CK) and zero-tillage was set as treatment (ZT). At the first year after applying zero-tillage, yield loss was observed in the ZT treatment which was attributed to the lower effective panicle number per area and grain number per panicle. However, from late season in 2018 to late season in 2019, significant higher grain yield was recorded in ZT than CK. ZT increased the net photosynthetic rate and chlorophyll content (SPAD value) by 6.81–20.77% and 7.23–23.80% in the last three cropping seasons compared with CK. Higher nitrogen, potassium and phosphorus accumulations in plant tissues were also recorded in ZT than CK from late season in 2018 to late season in 2019. Other hand, higher grain 2-AP content was recorded in ZT than CT in the whole six cropping seasons which might be related to the grain proline content. Furthermore, compared with CK, ZT significantly increased the soil organic matter content and the number of bacteria, fungi and actinomycetes in the last three cropping seasons. In conclusion, continuous zero-tillage could improve soil and increase the photosynthesis and nutrient accumulation and finally achieve the improvement of fragrant rice yield.

## Introduction

Fragrant rice is well known worldwide because of its unique ‘popcorn-like’ flavor and good grain quality. Among more than 200 volatile compounds in fragrant rice varieties, 2-acetyl-1-pyrroline (2-AP) was identified as the key component and it is now clearly established that the content of 2-AP represents the intensity of fragrant rice aroma^1,2^. In recent years, the demand and price of fragrant rice in the international market increased and thus, more farmers and scientists began to pay attention on how to improve the grain yield or the grain 2-AP content of fragrant rice in the field production^2^.

Land preparation is an important part during the field production of rice because tillage is required to reduce the losses of water and nutrients which caused by excessive percolation. Tillage also can reduce weeds and enhance nutrient availability^3^. However, previous study revealed that continual tillage will lead to the reductions of soil surface organic matter and soil poor structure and compaction^4^. Given the increasing rates of soil deterioration and soil erosion, more and more farmers and researchers have recently begun to pay attention to conservation tillage techniques^5^. Conservation tillage techniques are a series of sustainable agricultural measures which can reduce soil erosion, protect farmland ecological environments, and lead to coordinated development of ecological, benefits and social benefits via comprehensive supportive measures such as reduced tillage, no tillage, surface micro-topography transformation technology, surface cover and rational planting^5^. Normally, conservation tillage techniques include reduced tillage, no tillage, gentle slope contour tillage, furrow and ridge tillage, stubble mulching tillage, straw mulching and other farmland surface tillage technologies with their unique supporting machinery, respectively^6–8^. However, despite a lot of benefits of conservation tillage on the soil, some problems still exit in field production under conservation tillage conditions especially zero tillage. For example, Huang *et al*.^9^ revealed that nitrogen uptake of rice under zero-tillage condition was delayed during the early growth stages because of the inhibition of root growth which caused by the rhizospheric accumulation of inhibitory pseudomonads. Until now, the effect of zero-tillage on fragrant rice performance was rarely reported and explored.

Therefore, present study was conducted with a three-year field experiment in South China in order to investigate the influences of continuous zero-tillage on fragrant rice performance.

## Result

### Grain yield and yield related trails

There were some differences in grain yield and yield related attributes for both *Meixiangzhan-2* and *Xiangyaxiangzhan* between continuous zero-tillage and conventional tillage (Tables 1 and 2). In both early season and late season in 2017, lower grain yield, effective panicle number per area and grain number per panicle were recorded in ZT treatment than CK whilst in the last three cropping seasons in present study, compared with CK, ZT treatment significantly increased grain yield, effective panicle number per area and grain number per panicle. On the other hand, there was no remarkable difference between CK and ZT in seed-setting rate and 1000-grain weight in 2017, 2018 and 2019.

**Table 1:** Influence of continuous zero-tillage on grain yield and yield related attributes of fragrant rice “*Meixiangzhan-2*”.

Season	Treatment	Effective panicle number per m^2^	Grain number per panicle	Seed-setting rate (%)	1000-grain weight (g)	Grain yield (t ha^−1^)
**Early season in 2017**						
	CK	281.67a	142.82a	76.37a	19.87a	5.33a
	ZT	218.33b	131.49b	76.19a	19.72a	3.70b
**Late season in 2017**						
	CK	278.67a	138.63a	76.28a	19.50a	5.18a
	ZT	248.67b	127.98b	76.87a	19.78a	4.30b
**Early season in 2018**						
	CK	269.33a	141.61a	76.50a	20.27a	5.27a
	ZT	273.33a	136.51a	78.05a	20.24a	5.23a
**Late season in 2018**						
	CK	276.67b	148.15b	73.96a	20.12a	5.43b
	ZT	306.67a	158.35a	73.99a	19.33a	5.90a
**Early season in 2019**						
	CK	269.00b	145.32b	75.04a	20.10a	5.33b
	ZT	311.67a	152.47a	74.43a	19.75a	6.17a
**Late season in 2019**						
	CK	278.00b	142.60b	76.27a	20.19a	5.40b
	ZT	298.33a	158.09a	75.24a	20.29a	6.37a

The means in the same column followed by different lowercase letters for the same variety differ significantly at P < 0.05 according to the t-test. The same as applies below.

**Table 2:** Influence of continuous zero-tillage on grain yield and yield related attributes of fragrant rice “*Xiangyaxiangzhan*”.

Season	Treatment	Effective panicle number per m^2^	Grain number per panicle	Seed-setting rate (%)	1000-grain weight (g)	Grain yield (t ha^−1^)
**Early season in 2017**						
	CK	289.00a	137.94a	75.43a	19.68a	5.30a
	ZT	222.00b	132.85b	74.01a	19.14a	3.67b
**Late season in 2017**						
	CK	290.00b	144.19a	74.89a	21.02a	5.27a
	ZT	258.33a	128.53b	73.77a	20.18a	4.60b
**Early season in 2018**						
	CK	289.67a	144.17a	75.86a	19.60a	5.27a
	ZT	294.67a	147.54a	77.13a	20.59a	5.27a
**Late season in 2018**						
	CK	254.00b	140.22b	75.85a	19.48a	5.30b
	ZT	272.33a	146.58a	76.78a	19.50a	5.77a
**Early season in 2019**						
	CK	256.00b	146.45b	76.56a	20.58a	5.27b
	ZT	291.67a	161.06a	75.58a	19.90a	6.37a
Late season in 2019						
	CK	261.67b	147.42b	75.24a	20.89a	5.40b
	ZT	292.67a	159.74a	75.19a	19.68a	6.37a

### Net photosynthetic rate and SPAD values

As shown in Table 3, in both early season and late season in 2017, lower net photosynthetic rate was recorded in ZT than CK at tillering stage, heading stage and grain-filling stage for both *Meixiangzhan-2* and *Xiangyaxiangzhan*. Similar trend was also recorded in SPAD value. However, from late season in 2018 to late season in 2019, ZT treatment significantly increased the net photosynthetic rate and SPAD value compared with CK. For *Meixiangzhan-2*, ZT significantly increased the net photosynthetic rate and SPAD value by 6.81–18.46% and 7.69–21.84%; For *Xiangyaxiangzhan*, ZT significantly increased the net photosynthetic rate and SPAD value by 8.45–20.77% and 7.23–23.80%.

**Table 3:** Influence of continuous zero-tillage on net photosynthetic rate and SPAD values of fragrant rice.

Cultivar	Season	Treatment	Net photosynthetic rate	SPAD value				
			TS	HS	GS	TS	HS	GS
*Meixiangzhan-2*	Early season in 2017							
		CK	37.68a	36.96a	24.24a	38.80a	38.83a	25.81a
		ZT	25.92b	25.47b	16.65b	26.53b	26.63b	18.19b
	Late season in 2017							
		CK	37.21a	36.29a	23.14a	37.23a	38.01a	25.41a
		ZT	30.23b	30.86b	19.55b	30.89b	31.43b	21.18b
	Early season in 2018							
		CK	37.20a	37.14a	23.62a	38.97a	38.17a	25.75a
		ZT	36.25a	37.05a	23.31a	38.46a	38.19a	25.70a
	Late season in 2018							
		CK	38.82b	39.16b	24.51b	39.26b	39.28b	26.43b
		ZT	42.16a	41.83a	26.65a	43.66a	42.30a	29.28a
	Early season in 2019							
		CK	37.45b	37.58b	24.13b	38.98b	39.13b	26.05b
		ZT	43.47a	44.07a	27.35a	44.75a	44.95a	30.67a
	Late season in 2019							
		CK	38.14b	39.06b	24.48b	38.86b	40.10b	26.55b
		ZT	45.19a	43.99a	28.85a	47.35a	46.26a	31.55a
*Xiangyaxiangzhan*	Early season in 2017							
		CK	37.44a	36.82a	23.80a	38.66a	38.88a	25.90a
		ZT	25.99b	25.61b	16.40b	26.43b	26.82b	17.71b
	Late season in 2017							
		CK	36.62a	36.42a	23.38a	38.34a	39.20a	25.71a
		ZT	31.73b	32.26b	20.45b	32.87b	32.84b	22.73b
	Early season in 2018							
		CK	37.20a	36.86a	23.46a	38.15a	38.66a	25.43a
		ZT	36.74a	37.23a	23.71a	39.09a	38.31a	25.90a
	Late season in 2018							
		CK	37.31b	37.23b	23.74b	37.54b	37.87b	26.35b
		ZT	41.12a	41.19a	25.75a	41.16a	42.15a	28.26a
	Early season in 2019							
		CK	37.41b	37.48b	23.76b	38.24b	38.24b	25.86b
		ZT	44.11a	44.96a	28.37a	45.56a	47.35a	31.06a
	Late season in 2019							
		CK	37.86b	38.17b	24.30b	39.89b	39.18b	26.20b
		ZT	45.72a	44.73a	28.69a	45.06a	45.76a	31.39a

TS: Tillering stage; HS: Heading stage; GS: Grain-filling stage.

### Biomass accumulation

**Figure 1:**
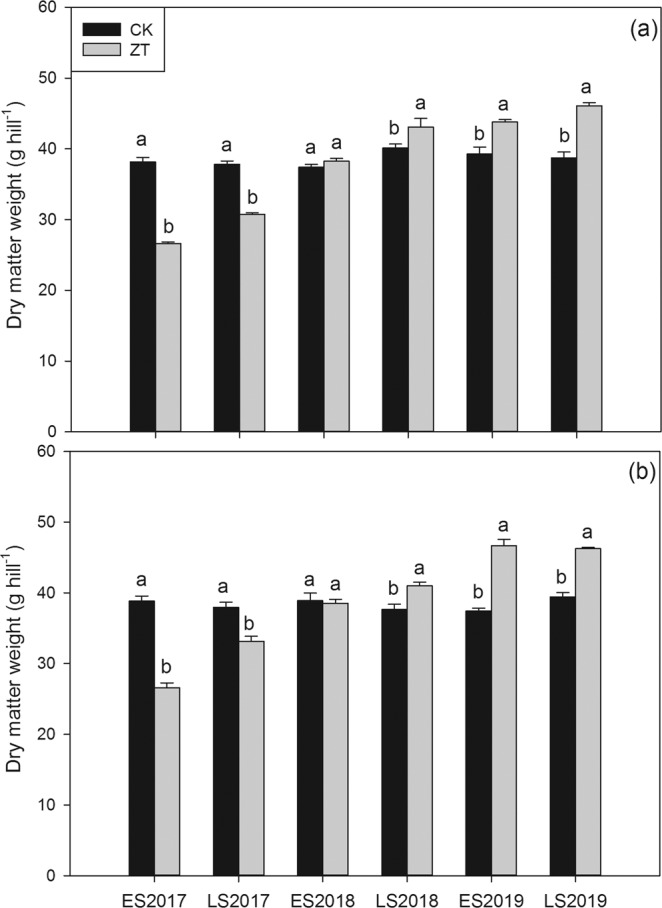
Influence of continuous zero-tillage on dry matter accumulation of fragrant rice. (**a**): *Meixiangzhan-2*; (**b**) *Xiangyaxiangzhan*; ES: Early season; LS: Late season; Capped bars represent S.E. of three replicates. Means sharing a common letter don’t differ significantly at (P ≤ 0.05) according to least significant difference (LSD) test for every season.

Continuous zero-tillage had impacts on in dry matter accumulation of fragrant rice (Fig. 1a,b). In early season and late season on 2017, Compared with CK, ZT treatment significantly decreased the dry matter weight by 30.15 and 18.70% for *Meixiangzhan-2* and by 31.49 and 12.62% for *Xiangyaxiangzhan*, respectively. However, in late season in 2018 and both early season and late season in 2019, ZT treatment significantly increased the dry matter weight at maturity by 7.33–19.09% for *Meixiangzhan-2* and by 8.86–24.65% for *Xiangyaxiangzhan*.

### Total nitrogen, phosphorus and potassium accumulation

**Figure 2:**
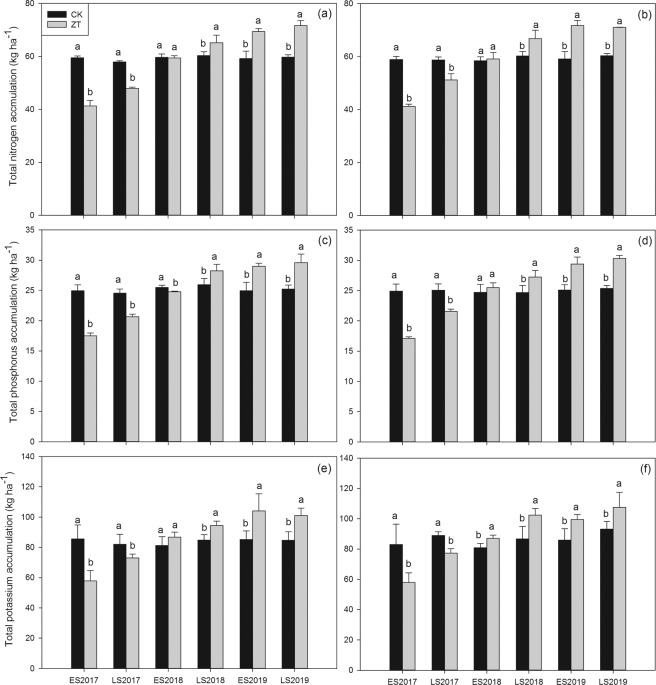
Influence of continuous zero-tillage on total nitrogen accumulation (**a,b**), total phosphorus accumulation (**c,d**) and total potassium accumulation (**e,f**) in aboveground tissue of fragrant rice. (**a,c,e**): *Meixiangzhan-2*; (**b,d,f**) *Xiangyaxiangzhan*;.

As shown in Fig. 2, continuous zero-tillage condition enhanced the absorption to nitrogen, phosphorus and potassium of fragrant rice. In both early season and late season in 2017, significant lower total nitrogen, phosphorus and potassium accumulations were both recorded in ZT treatment than CK. There was no significant difference between CK and ZT treatment on nutrient accumulations in early season in 2018. In the last three cropping seasons, significant higher total nitrogen, phosphorus and potassium accumulations were all recorded in ZT treatment than CK.

### Grain 2-AP content

**Figure 3:**
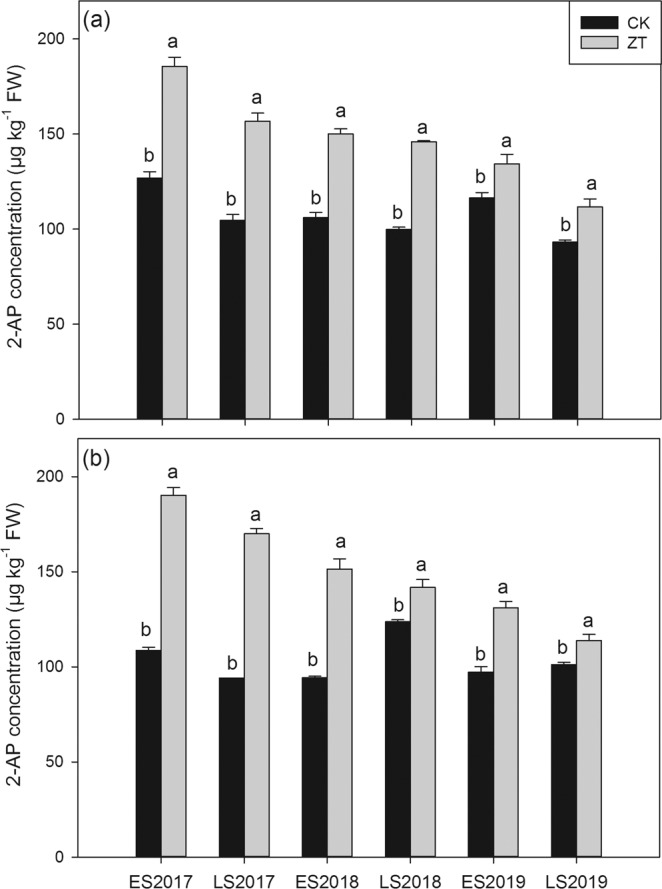
Influence of continuous zero-tillage on grain 2-AP content of fragrant rice. (**a**) *Meixiangzhan-2*; (**b**) *Xiangyaxiangzhan*;.

As shown in Fig. 3, ZT treatment significantly increased grain 2-AP content in fragrant rice in all six cropping seasons from 2017 to 2019. For *Meixiangzhan-2*, compared with CK, ZT treatment significantly increased grain 2-AP content by 46.30, 49.87, 41.53, 46.23, 15.36 and 19.92% in six planting season from 2017 to 2019, respectively; For *Xiangyaxiangzhan*, 75.00, 80.72, 60.74, 14.69, 34.70 and 12.39% higher 2-AP concentrations were recorded in ZT treatment than CK in six cropping season from 2017 to 2019, respectively.

### Grain proline content

**Figure 4:**
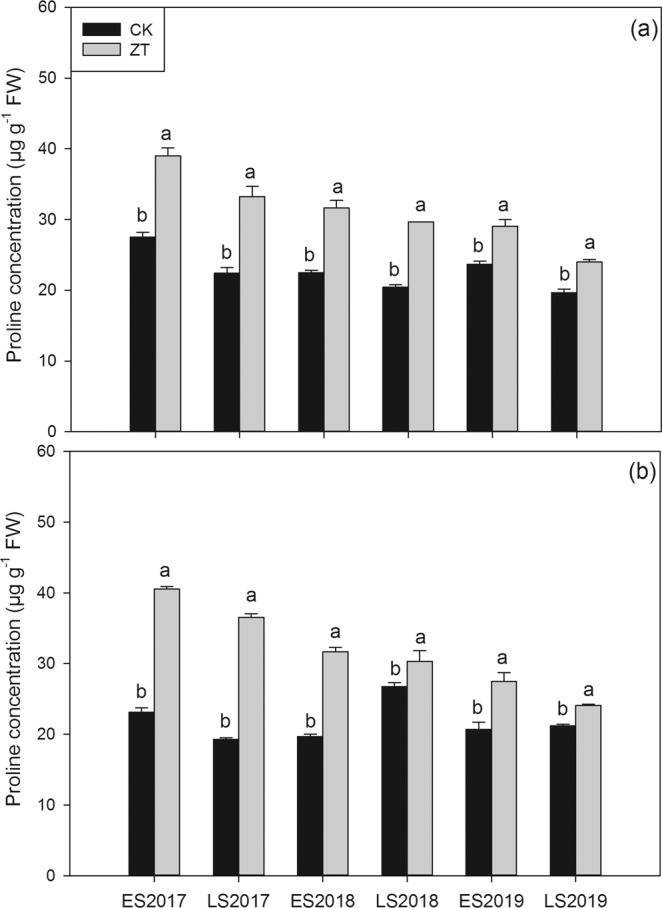
Influence of continuous zero-tillage on grain proline content of fragrant rice. (**a**) *Meixiangzhan-2*; (**b**) *Xiangyaxiangzhan*.

As shown in Fig. 4, ZT treatment significantly increased grain proline content in fragrant rice in all six cropping seasons from 2017 to 2019. For *Meixiangzhan-2*, compared with CK, ZT treatment significantly increased grain proline content by 41.88, 48.28, 40.73, 45.05, 22.76 and 22.23% in six planting season from 2017 to 2019, respectively; For *Xiangyaxiangzhan*, 75.08, 89.45, 60.81, 13.46, 32.89 and 13.73% higher proline concentrations were recorded in ZT than CK in six cropping seasons from 2017 to 2019, respectively.

### Soil organic matter and soil microorganism community quantity

**Figure 5:**
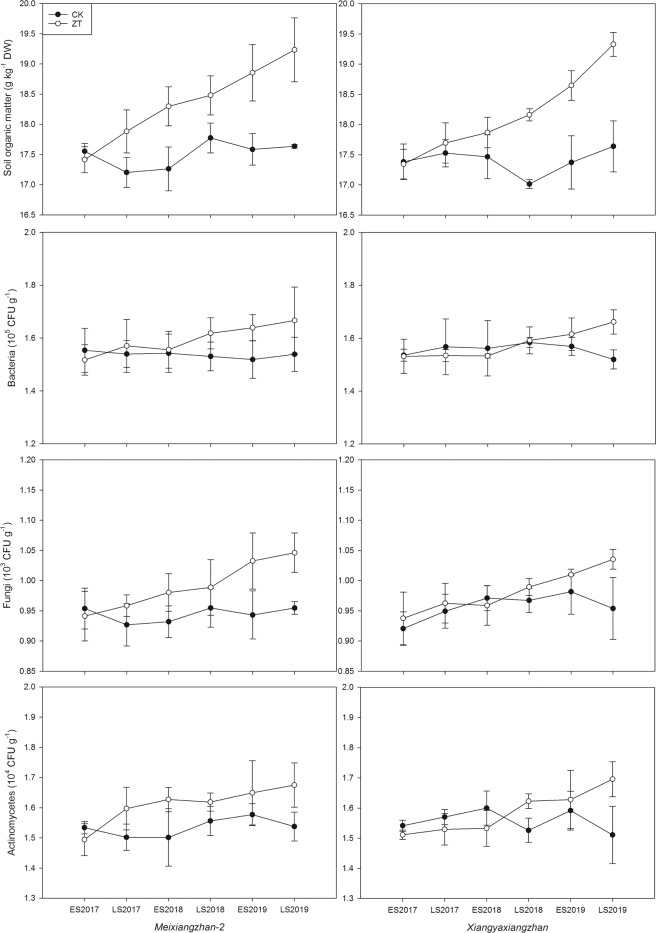
Influence of continuous zero-tillage on soil organic content and quantities of bacteria, fungi and actinomycetes.

As shown in Fig. 5, continuous zero tillage condition greatly improved the soil organic matter and soil microorganism community quantity in paddy field. For *Meixiangzhan-2*, the soil organic matter under ZT treatment was significantly higher than CK in all cropping seasons except early season in 2017. For *Xiangyaxiangzhan*, ZT treatment significantly increased soil organic matter compared to CK form late season in 2018 to late season in 2019. As far as soil microorganism communities were concerned, ZT treatment had higher quantities of bacteria, fungi and actinomycetes than CK from late season in 2018 to late season in 2019 for both fragrant rice cultivars.

### Correlation between grain yield and dry matter accumulation, nutrients accumulation, soil organic matter, soil microorganisms

As shown in Table 4, there exited significant and positive correlations between grain yield and dry matter weight, total nitrogen accumulation, total phosphorus accumulation, total potassium accumulation, soil organic matter, soil microorganisms (bacteria, fungi and actinomycetes). Similar trends were also observed in effective panicle number and grain number per panicle. On the other hand, neither seed-setting rate or 1000-grain weight have significant correlation with dry matter weight, total nitrogen accumulation, total phosphorus accumulation, total potassium accumulation, soil organic matter and soil microorganisms (bacteria, fungi and actinomycetes).

**Table 4:** Correlation analysis between grain yield and dry matter accumulation, nutrients accumulation, soil organic matter, soil microorganisms.

	Grain yield	Effective panicle number	Grain number per panicle	Seed setting rate	1000-grain weight
Dry matter weight	0.9948**	0.8147**	0.8940**	−0.1226	0.2145
Total N accumulation	0.9983**	0.8224**	0.8872**	−0.0911	0.2186
Total P accumulation	0.9953**	0.8144**	0.8815**	−0.0881	0.2382
Total K accumulation	0.9688**	0.7988**	0.8478**	−0.1298	0.2704
Soil organic matter content	0.7084*	0.5452*	0.7135*	−0.1807	0.0201
Bacteria	0.6873*	0.5686*	0.6663*	−0.1806	−0.0668
Fungi	0.6941*	0.5578*	0.7048*	−0.2315	−0.0211
Actinomycetes	0.6817*	0.5376*	0.6330*	−0.1128	−0.0109

## Discussion

Tillage is one of the factors which significantly influence the growth and development of rice. Present study revealed the effects of continuous zero tillage on yield formation and grain 2-AP content of fragrant rice under mechanized-transplanting double cropping rice system. Compared with conventional tillage, the yield loss occurred in the first two cropping seasons after the zero-tillage was applied (early season and late season in 2017). The decrement in grain yield was explained by the lower effective panicle umber, grain number per panicle, dry matter accumulation and total nitrogen, phosphorus, potassium accumulations. Our results were consistent with the previous study of Du^10^ who showed the zero tillage treatment would decrease the net photosynthetic rate and limit the growth and development of rice. Tillage is a process of soil puddling. Previous study showed that puddling in paddy field would be beneficial in preventing the loss of water and nutrients caused by excessive percolation^11^. Tillage also would create a softer environment for root growth and development while improving micro-nutrient availability^11^. Without the soil pudding effects caused by tillage, the structure of paddy soil would be easier for the nutrient loss which would cause the reduction in nutrient accumulation of fragrant rice under zero tillage conditions just as shown in present study. Our results agreed with the research of Du *et al*.^11^ who demonstrated that zero tillage would cause the yield loss by limiting the nitrogen uptake. Therefore, without benefits only came from the tillage, the physical and chemical properties of the soil might become less suitable for the growth and development of fragrant rice in the start cropping seasons of zero tillage and the nutrient loss and limit growth of root might be main reasons for the yield loss.

However, after three cropping seasons of continuous zero tillage, the grain yield of fragrant rice increased significantly compared with normal conventional tillage. At the most fundamental level, the increased grain yield under zero tillage treatment was attributed to the increment in effective panicle number per area and grain number per panicle. Compared with conventional tillage, continuous zero tillage condition significantly enhanced the nutrient (nitrogen, phosphorus and potassium) accumulation and dry matter weight in aboveground tissue of fragrant rice at maturity stage. Higher net photosynthetic rate and chlorophyll content were also recorded in ZT treatment than CK and those improvements were all beneficial for the yield formation of fragrant rice^12,13^. In addition, the results of present study showed that the continuous zero tillage condition significantly increased soil organic content as well as quantities of bacteria, fungi and actinomycetes. Previous studies have revealed that continuous zero tillage condition not only could improve the physical and chemical properties of soil, but also could enhance biological activity of soil because the small animal and microorganism in paddy field were allowed to grow and reproduce freely under zero tillage condition^14,15^. Abbas *et al*.^16^ also demonstrated that long term zero tillage could be a potential technology on promoting the productivity of cotton. Therefore, it could be concluded that continuous zero tillage improved the soil biological activities, then enhanced photosynthesis, nutrient accumulation and dry matter accumulation and finally increased grain yield of fragrant rice.

Interestingly, we observed that higher grain 2-AP concentration was recorded in ZT treatment than CK in all cropping seasons. The biosynthesis of 2-AP in fragrant rice is a very complicated bioprocess which affected by many factors. The study of Mo *et al*.^17^ depicted that shading stress during the grain filling stage would greatly up-regulate the grain 2-AP and proline concentrations in fragrant rice. Ren *et al*.^18^ demonstrated that application extra nitrogen fertilizer could increase the grain 2-AP and proline contents in fragrant rice. In our study, the trend of proline was similar as 2-AP which agreed with the study of Yoshihashi *et al*.^19^ who found that the proline was an important precursor of 2-AP. The increment in grain 2-AP concentration in 2017 might be attributed to abiotic stress because zero tillage will not only increase the bulk density of soil and result in nutrient loss, but also increase the hardness of soil and hinder the growth of root system as described by Du *et al*.^11^. But in 2018 and 2019, the increased 2-AP concentration might be explained by the higher nitrogen accumulation of fragrant rice under continuous zero tillage condition because 2-AP content is also associated with nitrogen dynamics in fragrant rice grain^20^. Furthermore, the increased soil organic matter also could be another reason for increased 2-AP but this guess needs to be verified because related study was rarely reported and explored.

## Conclusion

Continuous zero tillage condition increased effective panicle number per area, grain number per panicle and improved grain yield of fragrant rice but yield loss was caused in first two cropping seasons after the zero-tillage was applied. The changes in grain yield and yield related traits were attributed to the biomass and nutrient (nitrogen, phosphorus and potassium) accumulation. Zero tillage treatment also increased the grain 2-AP concentration of fragrant rice. The soil organic matter content and quantities of bacteria, fungi and actinomycetes in paddy field increased with the continuous zero tillage.

## Materials and Methods

### Experiment sites and plant materials

Seeds of two fragrant rice cultivars, *Meixiangzhan-2* (Lemont × Fengaozhan, bred by Rice research institute of Guangdong academy of agricultural sciences) and *Xiangyaxiangzhan* (Xiangsimiao126 × Xiangyaruanzhan, bred by Taishan institute of agricultural science), which are well known and widely grown in South China, provided by the College of Agriculture, South China Agricultural University, Guangzhou, China, were used as plant materials in present experiment. A three-year and six-season field experiment was conducted at Experimental Research Farm, College of Agriculture, South China Agricultural University, Zengcheng (23°13′N, 113°81′E, altitude 11 m), China. The experimental site enjoys a subtropical-monsoon climate. The experimental soil was sandy loam consisting of 15.06 g kg^−1^ organic matter, 1.10 g kg^−1^ total nitrogen, 53.72 mg kg^−1^ available nitrogen, 0.83 g kg^−1^ total phosphorus, 16.37 mg kg^−1^ available phosphorus, 11.19 mg kg^−1^ total potassium, and 120.08 mg kg^−1^ available potassium, and the pH was 6.56. Before sowing, the seeds were soaked in water for 12 h and then were transferred to a constant-temperature (36 °C) incubator for 12 h. Germinated seeds were sown in polyvinyl chloride (PVC) trays for nursery. Fifteen-day-old seedlings were transplanted into paddy field. In early season, fragrant rice cultivars were sown in March, transplanted in April and harvested in July; In late season, fragrant rice cultivars were sown in July, transplanted in August and harvested in November.

### Tillage treatment and crop management

Two tillage treatments were applied in present study and the description was as below:

CK: Conventional tillage followed as adopted by local farmers, twice puddling with a rotary cultivator before transplanting in each cropping season;

ZT: Zero tillage, no any tillage was done to the soil in paddy field before transplanting in each cropping season.

The experiment design was a strip plot design with two blocks. Each block was divided into six subplots. The area of each subplot was 90 m^2^ (3 × 30 m) while the same treatments were applied in the same blocks in each cropping season. The commercial compound fertilizer (YaraMila Fertilizer Company, China) was applied at the same amount of 105 kg·N·ha^−1^, 105 kg·P_2_O_5_·ha^−1^ and 105 kg·K_2_O·ha^−1^ with 60% as basal dose and 40% at tillering. The paddy field was flooded to about 3 cm water depth after the transplanting until the end of the tillering. Then, the water was drained for a week to control the production of infertile tillers and then a water later of 5–7 cm was kept during the grain-filling stage. All other agronomic practices i.e., pest and diseases management, and weed control were the same according the guidelines and standards recommended by the Tang *et al*.^21^.

### Determination of the 2-AP and proline content in grain at the maturity stage

Ten grams of fresh and mature grain samples were finely ground and then used for measurements of 2-AP via the simultaneous distillation-extraction method (SDE) to make a molar solution, which was then analyzed by a GCMS-QP 2010 Plus instrument (Shimadzu Corporation, Japan). The proline concentration was estimated according to the method described by Du *et al*.^11^.

### Measurements of yield and yield-related traits

At the maturity stage, the rice grains were harvested from a three-unit sampling area (1.00 m^2^) in each plot and then threshed by a machine according to methods descript be Du *et al*.^10^. The harvested grains dried in the sun, after which they were weighed to determine the grain yield. Twenty hills of rice from each plot were sampled to estimate the average effective panicle number per hill. Six representative hills of plants were then selected to estimate the yield-related traits.

### Estimation of biomass accumulation and total nutrient accumulation

At the maturity stage, rice plants of ten representative hill in each treatment were collected and dried out at 80°C for the measurement of dry matter weight and determination of total nitrogen, phosphorus and potassium accumulation in aboveground tissue according to the methods described by Pan *et al*.^22^. The rice plants collected were divided into leaf, stems and grain and then oven-dried at 80 °C, weighed, ground into powder for digestion. The digested samples were used to determine the total N content by the Kjeldahl method with a 2300 Kjeltec Analyser Unit (Foss Tecator AB, Swedish). The total P and K concentrations were determined by using the UV-VIS Spectrophotometer UV-2550 (SHIMADZU Corporation) and the Atomic Absorption Spectrophotometer AA-6300C (SHIMADZU Corporation) method, respectively.

### Determination of net photosynthetic rate and chlorophyll content

At tillering, heading and grain-filling stage, portable photosynthesis system (LI-6400, LI-COR, USA) was used to determine net photosynthetic rate at 09:00–10:30 a.m. with the following adjustments: photosynthetically active radiation at leaf surface was 1100 and 1200 μmol m^−2^ s^−1^, ambient CO_2_ concentration 380.0–400.0 μmol mol^−1^. SPAD meter ‘SPAD-502’ (Konica Minolta, Japan) was used for leaf chlorophyll contents.

### Determination of soil organic matter and soil microorganism community quantity

Soil samples from 0–20 cm depth were taken for the determination of soil organic matter and microorganisms by the 5-point sampling method at the maturity stage in each season and soil organic matter and soil microorganism community quantity was determinate according the methods described by Du *et al*.^11^. The light fraction (LF) and heavy fraction (HF) of soil organic matter were separated and the air-dried soil was homogenized with 30 mL NaI solution (gravity 1.8 g cm^−3^) in a 100 ml centrifuge tube by shaking on a reciprocating shaker for 60 min at 200 rpm, after which it was centrifuged at 1000 × g for 15 min. The LF, all floating material after centrifugation, was poured into a vacuum filter unit with a 0.45-μm nylon film, and the material retained by the film was washed with 0.01 M CaCl_2_ and distilled water. The HF remaining in the centrifuge tube was washed three times with ethanol to remove excess NaI, after which it was washed twice with distilled water. Next, the LF and HF were dried at 60 °C for 48 h, and then weighed and ground to pass through a 0.15-mm sieve for the organic determinations. the organic matter in LF and HF were determined by the wet oxidation method with K_2_Cr_2_O_7_ at 170–180 °C. The main soil microorganisms such as bacteria, fungi and actinomycetes were separated and counted by the dilution plate.

### Statistical analyses

The data was analyzed using the statistical software ‘Statistix 8.1’ (Analytical Software, Tallahassee, FL, USA), while differences among means were separated by using the least significant difference (LSD) test at the 5% probability level. Graphical representation was performed via Sigma Plot 14.0 (Systat Software Inc., California, USA).

## References

[1] Poonlaphdecha J (2016). Biosynthesis of 2-Acetyl-1-Pyrroline in Rice Calli Cultures: Demonstration of 1-Pyrroline as a Limiting Substrate. Food Chem..

[2] Luo, H. *et al*. Foliar Application of Sodium Selenate Induces Regulation in Yield Formation, Grain Quality Characters and 2-Acetyl-1-Pyrroline Biosynthesis in Fragrant Rice. *BMC Plant Biol*. **19** (2019). 10.1186/s12870-019-2104-4 PMC6858753 31730480

[3] Sharma, P. C. *et al*. Effect of Crop Management Practices on Crop Growth, Productivity and Profitability of Rice–Wheat System in Western Indo-Gangetic Plains. *Proceedings of the National Academy of Sciences, India Section B: Biological Sciences*. (2018).

[4] Chamen T (2016). Prevention Strategies for Field Traffic-Induced Subsoil Compaction: A Review: Part 2. Equipment and Field Practices. Soil. Res..

[5] Nawaz A, Farooq M, Lal R, Rehman A, Hafeez-ur-Rehman (2017). Comparison of Conventional and Conservation Rice-Wheat Systems in Punjab, Pakistan. Soil. Res..

[6] Wang Z, Lu L, Chen Q, Wen X, Liao Y (2016). Conservation Tillage Increases Soil Bacterial Diversity in the Dryland of Northern China. Agron. Sustain. Dev..

[7] Ma L (2019). Growth and Yield of Cotton as Affected by Different Straw Returning Modes with an Equivalent Carbon Input. Field crop. Res..

[8] Peigné J, Vian JF, Payet V, Saby N (2018). Soil Fertility After 10 Years of Conservation Tillage in Organic Farming. Soil. Res..

[9] Huang M, Chen J, Cao F, Jiang L, Zou Y (2016). Rhizosphere Processes Associated with the Poor Nutrient Uptake in No-Tillage Rice (Oryza Sativa L.) at Tillering Stage. Soil. Tillage Research..

[10] Du B (2018). Deep Fertilizer Placement Improves Rice Growth and Yield in Zero Tillage. Appl. Ecol. Env. Res..

[11] Du, P. *et al*. Different Tillage Induces Regulation in 2-Acetyl-1-Pyrroline Biosynthesis in Direct-Seeded Fragrant Rice. *BMC Plant Biol*. **19** (2019). 10.1186/s12870-019-1913-9 PMC6626333 31299895

[12] Zhou Y (2018). High Nitrogen Input Reduces Yield Loss From Low Temperature During the Seedling Stage in Early-Season Rice. Field crop. Res..

[13] Liu K (2019). Radiation Use Efficiency and Source-Sink Changes of Super Hybrid Rice Under Shade Stress During Grain-Filling Stage. Agron. J..

[14] Pandey LM, Pal S (2003). & Mruthyunjaya. Impact of Zero-Tillage Technology in the Rice (Oryza Sativa)-Wheat (Triticum Aestivum) System of Foothills of Uttaranchal State, India. Indian. J. Agr. Sci..

[15] Carr PM, Gramig GG, Liebig MA (2013). Impacts of Organic Zero Tillage Systems on Crops. Weeds, Soil. Quality. Sustainability-Basel..

[16] Abbas HG, Mahmood A, Ali Q (2016). Zero Tillage: A Potential Technology to Improve Cotton Yield. Genetika-Belgrade..

[17] Mo, Z. et al. Shading During the Grain Filling Period Increases 2-Acetyl-1-Pyrroline Content in Fragrant Rice. *Rice*. **8**, (2015). 10.1186/s12284-015-0040-y PMC4384914 25844114

[18] Ren Y (2017). Irrigation and Nitrogen Management Practices Affect Grain Yield and 2-Acetyl-1-Pyrroline Content in Aromatic. Rice. Appl. Ecol. Env. Res..

[19] Yoshihashi T, Huong NTT, Inatomi H (2002). Precursors of 2-Acetyl-1-pyrroline, a Potent Flavor Compound of an Aromatic Rice Variety. J. Agr. Food chem..

[20] Mo Z (2019). Regulations in 2-Acetyl-1-Pyrroline Contents in Fragrant Rice are Associated with Water-Nitrogen Dynamics and Plant Nutrient Contents. J. Cereal Sci..

[21] Xiangru, T. *et al*. Technical Regulations for Cultivation of Fragrant Rice (In Chinese). 5-7 (2014).

[22] Pan, S. et al. Effects of Nitrogen and Shading on Root Morphologies, Nutrient Accumulation, and Photosynthetic Parameters in Different Rice Genotypes. *Sci Rep-Uk*. **6** (2016). 10.1038/srep32148 PMC4997252 27557779

